# Comment on ‘Erector Spinae Plane block with fascial plane catheter for the Early Analgesia of Rib fractures in trauma (ESPEAR): a multicentre feasibility randomised trial’ (BJA Open 2025; 16: 100498)

**DOI:** 10.1016/j.bjao.2026.100557

**Published:** 2026-04-29

**Authors:** Joshua Lucas De Carvalho, Miruna-Maria Horga, Matt Beardmore

**Affiliations:** Department of Anaesthesia, Royal Cornwall Hospitals NHS Trust, Truro, UK

**Keywords:** chest wall injury, ESP, fascial plane catheter, major trauma, regional anaesthesia, rib fractures

Editor

We read with interest the findings of the Erector Spinae Plane block with fascial plane catheter for the Early Analgesia of Rib fractures in trauma (ESPEAR) feasibility trial by Hewson and colleagues.^1^ This trial thoroughly addressed core data points and made a concerted effort to meaningfully create a ‘placebo’ arm to scope future RCT design. We noted the low frequency of erector spinae plane (ESP) catheter (ESPc) insertions in the approximately 12-month recruitment period of the trial across three Major Trauma Centres, with just 3.6% (*n*=12) of potentially eligible screened patients having a fascial plane catheter sited. The main reasons for this were that (a) an alternative analgesic regimen was considered favourable and (b) lack of availability of appropriately trained and available clinician/researcher. Suggested modifications to future trial design were to study single-shot ESP blocks instead or control with multimodal analgesia only. Such low rates of ESPc insertion suggest a lack of belief in and/or inadequate pre-study training at the host institutions to reliably deliver the intended intervention. Abandoning the study of catheter-based ESP analgesia owing to these modifiable factors would be a mistake that risks delaying high-quality evidence of their potential benefits. We believe that ESPc analgesia is highly effective, safe, achievable by general anaesthetists and reduces potential harm from ‘multimodal’ (opiate) analgesia in patients with high-risk chest wall injury (CWI)—we share our experiences below.

In our trauma unit in Southwest England, we have been developing our CWI protocol and building our capacity to insert ESPc since 2017. In 2024, 359 patients were admitted for >24 h to our institution after blunt chest trauma, of which 90 patients had an ESPc sited (25.1% insertion rate) and were selected by a combination of Battle (STUdy of the Management of BLunt chest wall trauma) score,^2^ response to conventional analgesia, presence of key comorbidities, and clinician discretion. Bilateral and multilevel ESPcs are regularly practised instead of thoracic epidural analgesia. We have found the following benefits of prioritising ESPc over thoracic epidural analgesia: markedly reduced institutional barriers to establishing effective regional analgesia for these patients (e.g. access to higher care areas), facilitation of early mobilisation, ability to perform on patients with current therapeutic anticoagulation,^3^ and avoidance of complex venous thromboembolism prophylaxis considerations.

Of the ESPcs sited, 72.2% occurred ‘in-hours,’ and 27.8% occurred ‘out-of-hours,’ with a median time to insertion of 21.7 h from emergency department arrival. Average age of those undergoing ESPc and conventional opiate-based analgesia was 74 and 77, respectively. Our population includes a relatively larger cohort of frail, older patients who are unlikely to be accepted for Major Trauma Centre transfer or surgical rib fixation despite often extensive CWI. At least 51.1% of ESPc insertions involved resident doctors, either under the supervision of consultant anaesthetists or solo. Although there is a short learning curve to extending this Plan A block to include effective catheter insertion,^4^ we do not believe that this should be considered a ‘specialist’ intervention in any way. Furthermore, the additional time required to site a fascial plane catheter beyond the administration of a ‘single shot’ block is modest. Thus, catheter insertion is a more efficient use of limited resources, being a one-off intervention, and provides more consistent, prolonged analgesia for days afterwards. ESPc insertions occurred with full aseptic precautions in the emergency department, general ward areas, theatre recovery, and anaesthetic rooms. Thereafter, an intermittent bolus local anaesthetic regimen of 30 ml 0.25% bupivacaine 6-hourly for up to 5 days is administered by nurses in a standard ward setting. Of an estimated 500 ESPc inserted at our institution since 2017, the only significant complications identified among this closely audited cohort are one occurrence of chest wall haematoma (no intervention required) and one superficial infection (requiring surgical washout, confounding factors present).

A detailed local audit in 2022 showed that for isolated CWI, ESPcs were associated with a 4.5-day reduction in length of stay compared with conventional opiate-based regimens.^5^ It also showed markedly lower opiate consumption among those with the most severe CWI (CWI score >30). Notably, any patients with CWI receiving just a single dose of naloxone had an odds ratio for death of 7. We hypothesised that not only did ESPc provide superior analgesia and improve respiratory function and cough efficacy compared with opiate-based regimens but that the avoidance of opiate-related side effects also likely contributed to reduced harm. Consequently, we reduced our threshold to insert ESPc in those with evolving acute kidney injury, chronic kidney disease, bronchiectasis, and cardiac failure.

Since our baseline audit of patients with CWI in 2016 and the introduction of a care bundle for all these patients (consideration of regional analgesia, physiotherapy, early mobilisation, cohorting), mortality among this group has reduced from 11.6% to 4.8% (58.6% relative risk reduction). We believe that a significant contributor to this is our low threshold to site ESPc in patients with high-risk CWI. Although not formally case-matched data adjusted for relevant variables and vulnerable to numerous biases, our 2024 data showed 0% mortality among the ESPc group (*n*=90) *vs* 6.5% among those managed ‘conservatively’ (*n*=269). This may signal a paradigm shift away from accepting opiate-based regimens as a ‘norm’ for patients with higher-risk CWI. Although the latter is often convenient and less resource intensive, this approach may carry significant harmful side effects or even increase mortality, and we now have superior alternatives readily available.

Thus, we have demonstrated over several years the analgesic efficacy of ESPc and the achievement of sufficiently high competency rates among general anaesthetists to promptly deliver this intervention. We welcome the interest in scientifically establishing the efficacy of ESPc analgesia regimens beyond individual institutions and note that we would potentially meet the potential future trial site requirements given that ‘ESP catheter techniques are already standard care’ in our institution.^1^ However, the measurable and clinically apparent beneficial effects demonstrated locally from ESPc would give us misgivings about equipoise when considering including a placebo ESPc group. Our current direction of travel is, in parallel to promptly listing patients for ESPc, to extend provision of single-shot ESP blocks to emergency physicians^6^—the ‘fascia iliaca of the chest’, if you will—as a bridge to ‘in-hours’ ESPc insertion by anaesthetists, thus minimising higher-risk CWI patients’ exposure to opiates and ensuring optimal early analgesia.

## Declaration of interest

The authors declare that they have no conflicts of interest.
